# The Mitochondrial Pyruvate Carrier Coupling Glycolysis and the Tricarboxylic Acid Cycle Is Required for the Asexual Reproduction of Toxoplasma gondii

**DOI:** 10.1128/spectrum.05043-22

**Published:** 2023-03-15

**Authors:** Congcong Lyu, Yukun Chen, Yanan Meng, Jichao Yang, Shu Ye, Zhipeng Niu, Issam EI-Debs, Nishith Gupta, Bang Shen

**Affiliations:** a State Key Laboratory of Agricultural Microbiology, College of Veterinary Medicine, Huazhong Agricultural University, Wuhan, Hubei Province, People’s Republic of China; b Hubei Hongshan Laboratory, Wuhan, Hubei Province, People’s Republic of China; c Shenzhen Institute of Nutrition and Health, Huazhong Agricultural University, Shenzhen, Guangdong Province, People’s Republic of China; d Department of Molecular Parasitology, Faculty of Life Sciences, Humboldt University, Berlin, Germany; e Intracellular Parasite Education and Research Labs (iPEARL), Department of Biological Sciences, Birla Institute of Technology and Science, Pilani (BITS-P), Hyderabad, India; f Shenzhen Branch, Guangdong Laboratory for Lingnan Modern Agriculture, Genome Analysis Laboratory of the Ministry of Agriculture, Agricultural Genomics Institute at Shenzhen, Chinese Academy of Agricultural Sciences, Shenzhen, Guangdong Province, People’s Republic of China; Hubei University of Medicine

**Keywords:** mitochondrial pyruvate carrier, glycolysis, TCA cycle, branched-chain ketoacid dehydrogenase, acetyl-CoA

## Abstract

Toxoplasma gondii is an obligate intracellular parasite capable of infecting humans and animals. The organism has extraordinary metabolic resilience that allows it to establish parasitism in varied nutritional milieus of diverse host cells. Our earlier work has shown that, despite flexibility in the usage of glucose and glutamine as the major carbon precursors, the production of pyruvate by glycolytic enzymes is central to the parasite’s growth. Pyruvate is metabolized in a number of subcellular compartments, including the mitochondrion, apicoplast, and cytosol. With the objective of examining the mechanism and importance of the mitochondrial pool of pyruvate imported from the cytosol, we identified the conserved mitochondrial pyruvate carrier (MPC) complex, consisting of two subunits, MPC1 and MPC2, in T. gondii. The two parasite proteins could complement a yeast mutant deficient in growth on leucine and valine. Genetic ablation of either one or both subunits reduced the parasite’s growth, mimicking the deletion of branched-chain ketoacid dehydrogenase (BCKDH), which has been reported to convert pyruvate into acetyl-coenzyme A (CoA) in the mitochondrion. Metabolic labeling of the MPC mutants by isotopic glucose revealed impaired synthesis of acetyl-CoA, correlating with a global decrease in carbon flux through glycolysis and the tricarboxylic acid (TCA) cycle. Disruption of MPC proteins exerted only a modest effect on the parasite’s virulence in mice, further highlighting its metabolic flexibility. In brief, our work reveals the *modus operandi* of pyruvate transport from the cytosol to the mitochondrion in the parasite, providing the missing link between glycolysis and the TCA cycle in T. gondii.

**IMPORTANCE** T. gondii is a zoonotic parasite capable of infecting many warm-blooded organisms, including humans. Among others, a feature that allows it to parasitize multiple hosts is its exceptional metabolic plasticity. Although T. gondii can utilize different carbon sources, pyruvate homeostasis is critical for parasite growth. Pyruvate is produced primarily in the cytosol but metabolized in other organelles, such as the mitochondrion and apicoplast. The mechanism of import and physiological significance of pyruvate in these organelles remains unclear. Here, we identified the transporter of cytosol-derived pyruvate into the mitochondrion and studied its constituent subunits and their relevance. Our results show that cytosolic pyruvate is a major source of acetyl-CoA in the mitochondrion and that the mitochondrial pyruvate transporter is needed for optimal parasite growth. The mutants lacking the transporter are viable and virulent in a mouse model, underscoring the metabolic plasticity in the parasite’s mitochondrion.

## INTRODUCTION

Toxoplasma gondii is a zoonotic pathogen of the protozoan phylum Apicomplexa, which contains many parasites that cause serious diseases in humans and animals (1, 2). T. gondii infection is widely distributed, and the worldwide seroprevalence in the human population is nearly 30% (2, 3). It can cause abortions and stillbirth in prenatal animals and women (1, 2). The parasite has a complex natural life cycle that consists of multiple developmental stages in primary (sexual) and secondary (intermediate) host organisms. Notably, Toxoplasma can be transmitted between intermediate hosts, contributing to its high prevalence. For example, a common route for human infection is the consumption of undercooked meat containing viable encysted parasites (4). The parasite’s ability to interconvert between the acute (tachyzoite) and chronic (bradyzoite) infectious stages underlies the widespread transmission between intermediate hosts (5).

T. gondii has evolved sophisticated mechanisms to survive in an extremely broad range of host cells with distinct nutritional environments. Indeed, a series of studies has demonstrated profound metabolic flexibilities of its tachyzoite stage. For example, the parasite encodes a type II fatty acid synthesis (FASII) pathway in the apicoplast (a chloroplast relict), as well as a fatty acid elongation route in the endoplasmic reticulum for making very-long-chain and unsaturated fatty acids (6–8). Additionally, it is capable of scavenging host-derived acyl chains (9). Such flexibility in fatty acid acquisition renders *de novo* synthesis (FASII) and salvage pathways nonessential for the parasite’s survival. Recently, Krishnan et al. reported a metabolic model of tachyzoites using a PhenoMapping workflow (10), predicting 26 pairs of synthetic lethal genes that further highlight the unprecedented metabolic plasticity in T. gondii.

The metabolic resilience of tachyzoites is also reflected in their central carbon metabolism. Although glucose is the preferred carbon source, parasite mutants lacking the major glucose transporter GT1 exhibit only a mild growth defect *in vitro* and are fully virulent *in vivo* (11). When glucose utilization is impaired in mutants lacking GT1 or hexokinase, glutamine can be efficiently deployed as a substitute carbon source by tachyzoites (12). Glutamine is first converted to glutamate and then to alpha-ketoglutarate to enter the mitochondrial tricarboxylic acid (TCA) cycle. It can also be metabolized via glutamate decarboxylase to gamma-aminobutyric acid (GABA) (13), which is shuttled into the mitochondrion and converted to succinate, feeding the TCA cycle. Albeit the GABA shunt is not essential for parasite growth or virulence under standard culture conditions, it is an important energy source when glucose becomes limited (13). When glutamine is the only major carbon source, it is not only catabolized via the TCA cycle to generate ATP but also utilized to produce glycolytic intermediates, some of which are precursors for macromolecular synthesis. In the absence of GT1, the mitochondrial isoform of phosphoenolpyruvate carboxykinase (PEPCK) that converts oxaloacetate into phosphoenolpyruvate (PEP) becomes essential for the parasite’s survival and growth (14). Oxaloacetate derived from glutamine can replenish glycolytic intermediates by PEP synthesis and gluconeogenesis. In other words, GT1 and PEPCK are synthetic lethal in tachyzoites.

Many glycolytic enzymes in T. gondii have two or more isoforms, which are either differentially expressed during the life cycle (such as bradyzoite-specific enolase 1 and tachyzoite-specific enolase 2), have different catalytic mechanisms (the ATP-dependent phosphofructokinase 1 [PFK1] and the pyrophosphate-dependent phosphofructokinase 2 [PFK2]), or show distinct subcellular localization (pyruvate kinase 1 [PYK1] in the cytosol and PYK2 in the apicoplast) (15–19). Some of these enzymes are required for optimal propagation of tachyzoites; for instance, depletion of PYK1 or PFK2 severely compromises the parasite’s growth and virulence (18, 20). In particular, mutagenesis of PYK1 and PYK2 suggests a critical role of pyruvate homeostasis in tachyzoite proliferation. PYK1 is needed for pyruvate production and parasite growth, irrespective of the carbon source (glucose or glutamine). Structurally similar compounds like lactate and alanine can be converted to pyruvate and partly restore the growth defect of the PYK1-depleted mutants (20).

Pyruvate is utilized by several metabolic pathways in multiple organelles. While the PYK1-derived cytosolic pool of pyruvate is crucial for tachyzoites, the underlying cause is unclear. It can be converted to lactate and amino acids in the cytosol and/or imported into the mitochondrion, producing acetyl-coenzyme A (CoA) for the TCA cycle. The latter reaction is catalyzed by a branched-chain ketoacid dehydrogenase (BCKDH) enzyme (21), which has been repurposed to serve the catalytic role of pyruvate dehydrogenase (PDH) in apicomplexan parasites. The cytosolic pyruvate may also enter the apicoplast to fuel the methylerythritol 4-phosphate (MEP) pathway for producing isoprenoids, as well as the FASII pathway for fatty acid synthesis. Because the apicoplast-localized PYK2 is dispensable even though the MEP and FASII pathways are required for tachyzoite growth, it is reasonable to assume that the organelle imports pyruvate from the cytosol. Likewise, we postulate the presence of a transport system in the parasite mitochondrion, which constituted the focus of this study of the tachyzoite stage of T. gondii.

## RESULTS

### T. gondii encodes two functional MPC proteins.

**FIG 1:**
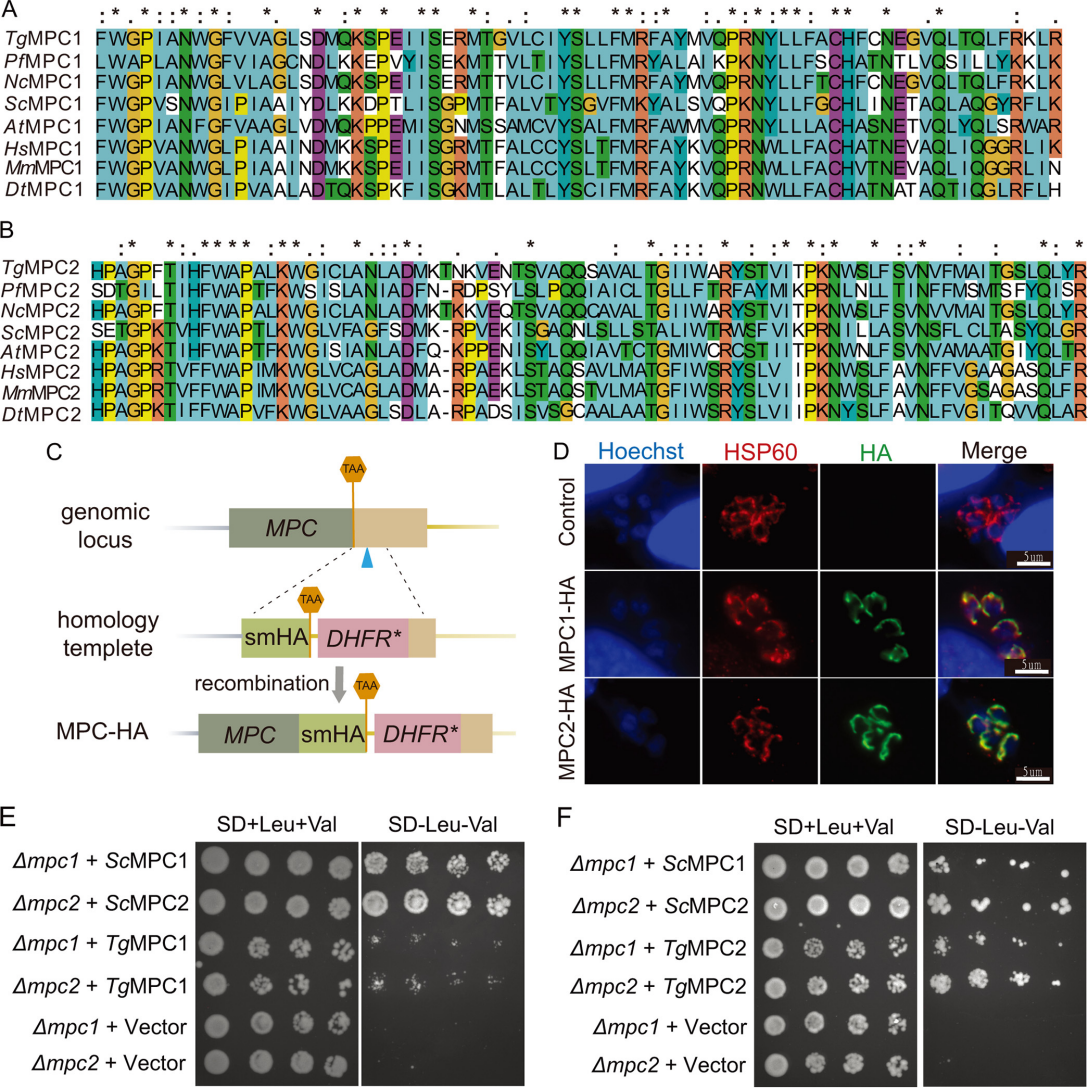
Toxoplasma expresses two MPC subunits in the mitochondrion. (A and B) Sequence alignment of Toxoplasma MPC1 (A) and MPC2 (B) with corresponding MPC subunits from selected organisms. Sequences at the conserved regions are shown. The alignments were performed using Clustal X2. (C) *Tg*MPC1 or *Tg*MPC2 subunits were C-terminally tagged with the spaghetti monster tag containing 10 HA epitopes (smHA) at the endogenous gene locus through CRISPR/Cas9-mediated site-specific gene insertion in the RH Δ*ku80* strain. The blue arrowhead indicates the CRISPR cut site. (D) Immunofluorescence assays of the 10 HA-tagged strains constructed as shown in panel C to check the localization of MPC1 and MPC2. *Tg*HSP60 was used as a mitochondrion-specific marker for colocalization purposes. (E and F) Serial dilutions of the indicated yeast strains were spotted on plates with (+) or without (−) valine and leucine and grown at 30°C for 3 days.

To search for the mitochondrial pyruvate carrier (MPC) subunits in T. gondii, *bona fide* MPCs from humans and yeasts were used as the query sequences for BLAST analyses in ToxoDB (https://toxodb.org). The top hits were used as baits for reciprocal BLAST searches in human and yeast genomes. We found two orthologs in Toxoplasma, termed MPC1 (TGGT1_235880) and MPC2 (TGGT1_204370). The open reading frames of T. gondii MPC1 (*Tg*MPC1) and *Tg*MPC2 encode relatively small proteins of 160 and 135 amino acids, respectively. Their sequences are well conserved with respect to the corresponding MPC subunits from other eukaryotes, as suggested by multiple sequence alignment (Fig. 1A and B). To assess the subcellular localization of *Tg*MPCs, a spaghetti monster-hemagglutinin (smHA) epitope tag (22) was fused to their C-terminal ends using CRISPR/Cas9-mediated homologous recombination at the 3′ end of the target gene (Fig. 1C) (23). *Tg*MPC1-smHA and *Tg*MPC2-smHA colocalized with a mitochondrial marker, T. gondii heat shock protein 60 (*Tg*HSP60), in transgenic parasites (Fig. 1D).

Next, we tested the function of *Tg*MPC1 and *Tg*MPC2 by expressing them in a Saccharomyces cerevisiae yeast mutant lacking its endogenous MPC1 (*ScΔmpc1*) or MPC2 (*ScΔmpc2*) (24). As shown by the results in Fig. 1E and F, the *ScΔmpc1* and *ScΔmpc2* strains were auxotrophic for leucine and valine, which serve as carbon sources in the absence of mitochondrial pyruvate transport. The growth of both mutants could be restored by ectopic expression of the corresponding yeast proteins (*Sc*MPC1 and *Sc*MPC2; positive controls), whereas the empty-vector negative control was unable to rescue the auxotrophic behavior of either strain for branched-chain amino acids (BCAAs). The expression of *Tg*MPC1 partly complemented the growth of *ScΔmpc1* and *ScΔmpc2* mutants on plates without valine and leucine, while *Tg*MPC2 conferred a complete restoration of the growth of *ScΔmpc1* and *ScΔmpc2* mutants. We also performed functional complementation assays in defined liquid cultures without or with BCAAs, confirming our results (Fig. S1 in the supplemental material).

### MPC1 and/or MPC2 is dispensable for parasite growth but required for optimal parasite growth.

**FIG 2:**
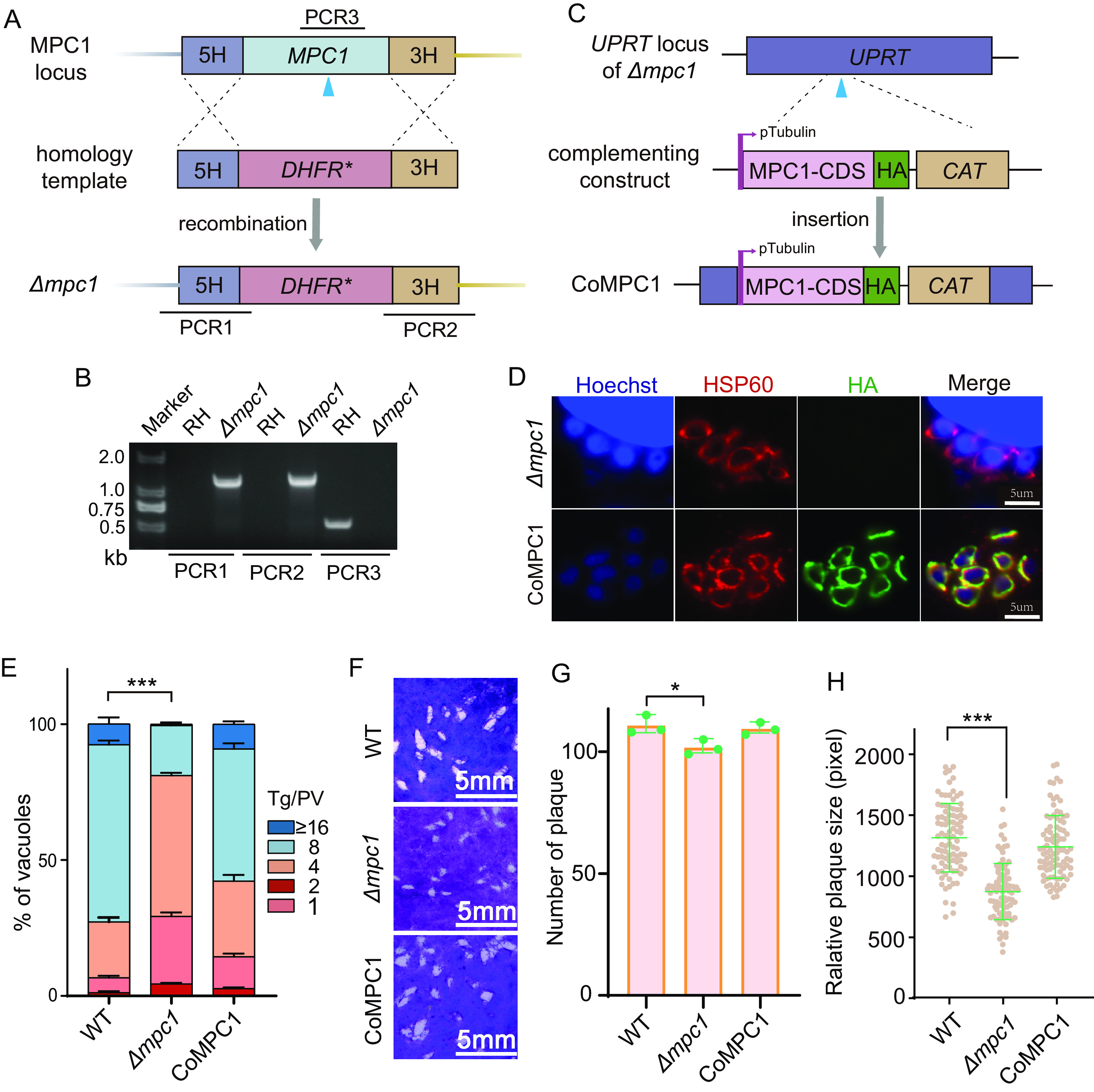
Generation and characterization of a Δ*mpc1* mutant. (A) Schematic illustration of the replacement of the *MPC1* gene with the DHFR* selection marker, using CRISPR/Cas9-assisted homologous recombination. The blue arrowhead indicates the CRISPR cut site, and PCR1, PCR2, and PCR3 denote the diagnostic PCRs used for identification of Δ*mpc1* clones. (B) Diagnostic PCRs of a Δ*mpc1* clone. The parental strain, RH, was included as a control. (C) Strategy for inserting an HA-tagged, MPC1-expressing cassette into the *UPRT* locus of the Δ*mpc1* mutant to construct the complementation strain CoMPC1. The chloramphenicol resistance marker *CAT* (chloramphenicol acetyl transferase) was used for selection. (D) Immunofluorescence assay checking the expression of complementing MPC1 in the CoMPC1 strain. The Δ*mpc1* mutant was used as a control. (E) Size distribution of the parasitophorous vacuoles (PV) after 24 h of replication in HFF cells. The mean values ± SEM from three independent experiments are shown. Significance was determined using two-way ANOVA. ***, *P ≤ *0.001. (F) A 7-day plaque assay comparing the overall growth of the indicated strains. (G and H) The numbers and relative sizes of plaques in the experiment whose results are shown in panel F. The mean values ± SD of more than 90 plaques are graphed. Significance was determined using Student’s *t* test. *, *P* ≤ 0.05; ***, *P* ≤ 0.001.

To evaluate the physiological significance of the *Tg*MPCs in T. gondii, we first deleted the *MPC1* gene by replacing it with a pyrimethamine-resistant DHFR*, a mutant version of Toxoplasma dihydrofolate reductase (Fig. 2A) (23). The resulting parasite clones were screened by diagnostic PCRs (PCR1, PCR2, and PCR3) (Fig. 2B). We also constructed a complemented strain expressing MPC1 (CoMPC1) in the deletion mutant to facilitate subsequent phenotypic analyses. To do this, an HA-tagged *Tg*MPC1 expression cassette was inserted into the *UPRT* locus of the mutant (Fig. 2C). The protein localized in the mitochondrion, as expected (Fig. 2D). The Δ*mpc1* mutant proliferated more slowly than the parental strain, and complementation by ectopic MPC1 restored the replication defect (Fig. 2E). Consequently, the plaques formed by the knockout strain were much smaller than the plaques formed by the parental and CoMPC1 strains (Fig. 2F to H). These results demonstrated a need for MPC1 for tachyzoite growth under standard culture conditions.

**FIG 3:**
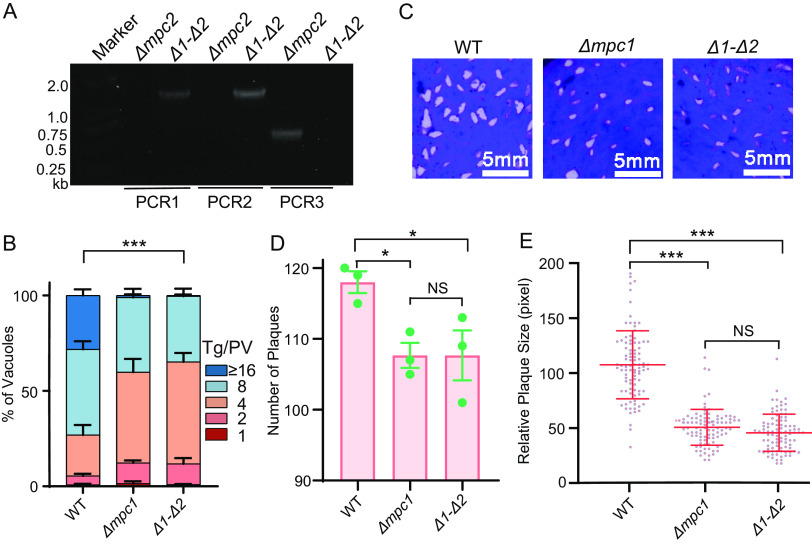
Mutants lacking both MPC1 and MPC2 displayed growth defects similar to that of the Δ*mpc1* mutant. (A) Diagnostic PCRs of the Δ*mpc1*-Δ*mpc2* double-deletion mutant (Δ*1-Δ2*), which was constructed by deleting MPC1 in Δ*mpc2*, using the same strategy depicted in Fig. 2A. (B) Intracellular replication assays of indicated strains, as done in the experiment whose results are shown in Fig. 2E. The mean values ± SEM from three independent experiments are shown. Significance was determined using two-way ANOVA. ***, *P* ≤ 0.001. (C to E) Plaque assays comparing the growth of Δ*mpc1* and Δ*mpc1*-Δ*mpc2* mutants to that of RH, as performed in the experiments whose results are shown in Fig. 2F to H. The mean values ± SD are shown. Significance was determined using Student’s *t* test. *, *P* ≤ 0.05; ***, *P* ≤ 0.001; NS, not significant.

Studies of other organisms suggest that functional MPC comprises two subunits in a heterodimeric complex. However, human MPC2 has also been reported to form a homodimeric complex (25). We, therefore, tested the impact of MPC2 inactivation on parasite growth. A Δ*mpc2* single mutant was generated, and its plaque growth and replication compared to those of the parental strain (Fig. S2). It displayed a defect similar to that of the Δ*mpc1* mutant. To further test whether the loss of both subunits would have additive effects, we constructed a Δ*mpc1-Δmpc2* double mutant (Δ*1-Δ2*) by deleting *Tg*MPC1 from the Δ*mpc2* strain. The recombination-specific screening PCRs (similar in design to those depicted in Fig. 2A and B) confirmed the deletion of *Tg*MPC1 in a Δ*1-Δ2* clone (Fig. 3A). The growth assays suggested that the Δ*1-Δ2* double mutant phenocopied each MPC single mutant (Δ*mpc1* or Δ*mpc2*) (Fig. 3B to E and Fig. S2C to E). These results suggest that *Tg*MPC1 and *Tg*MPC2 are not functionally redundant but may instead function as a heterodimeric complex.

### The MPC deletion strains mimic the BCKDH-deficient mutants.

**FIG 4:**
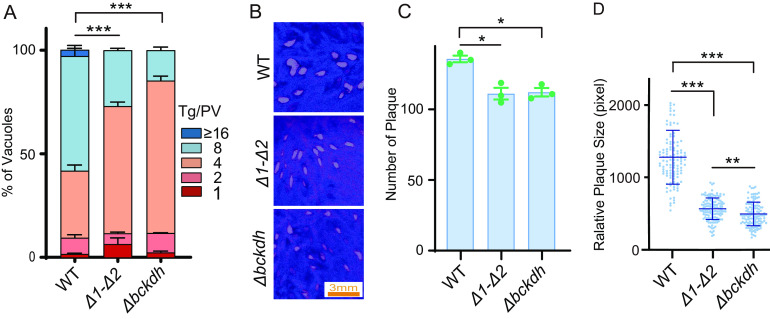
MPC-deficient parasites had growth defects similar to that of the Δ*bckdh* mutant. (A) Intracellular replication assays comparing the proliferation rates of the indicated strains in HFF, as done in the experiment whose results are shown in Fig. 2E. The mean values ± SEM from three independent experiments are shown. Significance was determined using two-way ANOVA. ***, *P < *0.001. (B to D) Plaque assays of indicated strains, performed as in the experiments whose results are shown in Fig. 2F to H. The mean values ± SD are shown. Significance was determined using Student’s *t* test. *, *P* ≤ 0.05; **, *P* ≤ 0.01; ***, *P* ≤ 0.001.

*Tg*BCKDH converts pyruvate to acetyl-CoA in the parasite’s mitochondrion (21). Therefore, we reasoned that if the glycolysis-derived pyruvate imported by MPC proteins was the only source for acetyl-CoA synthesis in the mitochondrion, a mutant with their deletion should mimic the growth of the BCKDH mutant. To test this notion, we generated a Δ*bckdh* mutant by replacing the corresponding gene with DHFR*, using a strategy similar to the one depicted in Fig. 2A. Indeed, the Δ*bckdh* and Δ*1-Δ2* strains exhibited nearly identical growth patterns in the plaque and replication assays (Fig. 4). In yeast and mammalian cells, BCAAs like leucine and valine can serve as a source of acetyl-CoA and thereby render MPC proteins dispensable (Fig. 1E and Fig. S1). To examine whether the degradation of BCAAs has any role in acetyl-CoA production in mutants lacking MPC, we targeted the branched-chain aminotransferase (*BCAT*) that was required for BCAA degradation and constructed a Δ*mpc2-Δbcat* double mutant (Fig. S2B). Surprisingly, the growth of the Δ*mpc2-Δbcat* mutant was similar to that of the MPC mutants (Fig. 3B to E). These results suggest that an interrupted supply of pyruvate in the mitochondrion cannot be compensated by the degradation of BCAAs and that pyruvate imported by MPC is a major source of mitochondrial acetyl-CoA.

### Deletion of MPC impairs the central carbon metabolism of tachyzoites.

**FIG 5:**
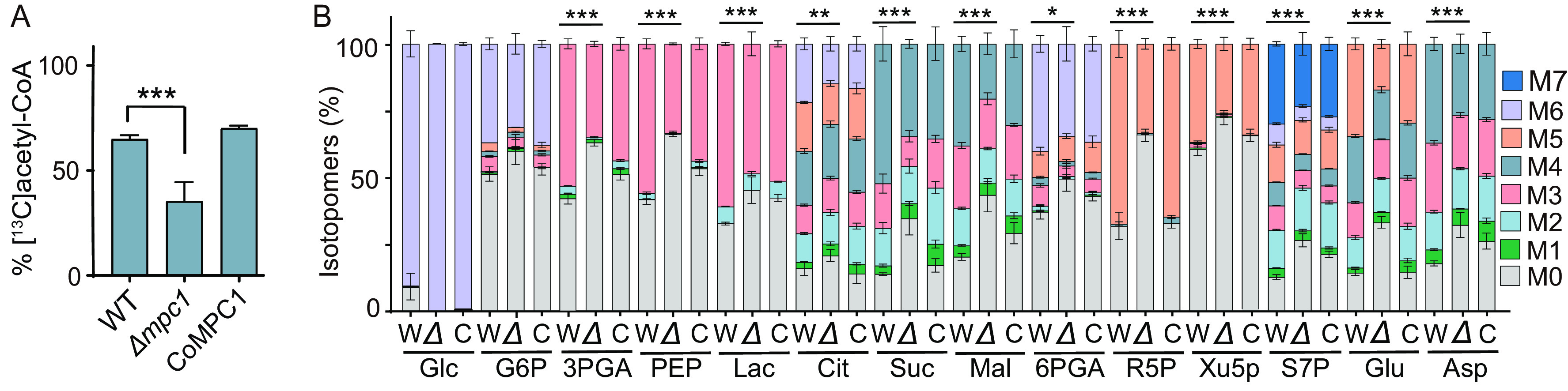
Alteration of central carbon metabolism in MPC1-deficient mutants. Purified tachyzoites of the indicated strains (W, wild-type strain RH; Δ, Δ*mpc1*; C, CoMPC1) were labeled with [^13^C_6_]glucose for 4 h at the extracellular stage, and the incorporation of ^13^C into metabolites was determined by LC-MS. (A) Percentages of acetyl-CoA that were labeled with ^13^C. The mean values ± SEM from three independent experiments are shown. Significance was determined using Student’s *t* test. ***, *P* ≤ 0.001. (B) ^13^C inclusion in each isotopologue of the indicated metabolites. M0 to M7 indicate the number of ^13^C atoms in each compound. The mean values ± SEM from three independent experiments are shown. Significance was determined using two-way ANOVA. *, *P* ≤ 0.05; **, *P* ≤ 0.01; ***, *P* ≤ 0.001. Glc, glucose; G6P, glucose 6-phosphate; 3PGA, 3-phosphoglyceric acid; PEP, phosphoenolpyruvic acid; Lac, lactate; Cit, citrate; Suc, succinate; Mal, malate; 6PGA, 6-phosphogluconic acid; R5P, ribose 5-phosphate; Xu5P, xylulose-5-phosphate; S7P, sedoheptulose 7-phosphate; Glu, glutamic acid; Asp, aspartic acid.

To explore the mechanisms underlying the growth defect observed in the MPC mutants, we investigated metabolic changes in MPC1 knockout tachyzoites. We first examined the efficiency of acetyl-CoA production from glucose by labeling parasites with [^13^C_6_]glucose for 4 h, followed by liquid chromatography-mass spectrometry (LC-MS) (Fig. 5A). In the parental RH parasites, >60% of the total acetyl-CoA pool was labeled with ^13^C (with one or more carbons being ^13^C), which, however, was reduced to about 30% in the Δ*mpc1* mutant. The decrease of acetyl-CoA labeling was restored in the MPC1-complemented strain. We also tested the impact of MPC1 mutation on the carbon flux via glycolysis, the TCA cycle, and the pentose phosphate pathway (PPP). Tachyzoites were labeled with [^13^C_6_]glucose, and the inclusion of ^13^C into key metabolites was quantified. The Δ*mpc1* strain displayed impaired flux of glucose-derived carbon into several metabolic intermediates, including PEP, lactate, citrate, malate, succinate, and ribose-5-phosphate. As expected, MPC1 complementation restored the observed metabolic phenotype (Fig. 5B).

### The MPC and BCKDH mutants show attenuated virulence in mice.

**FIG 6:**
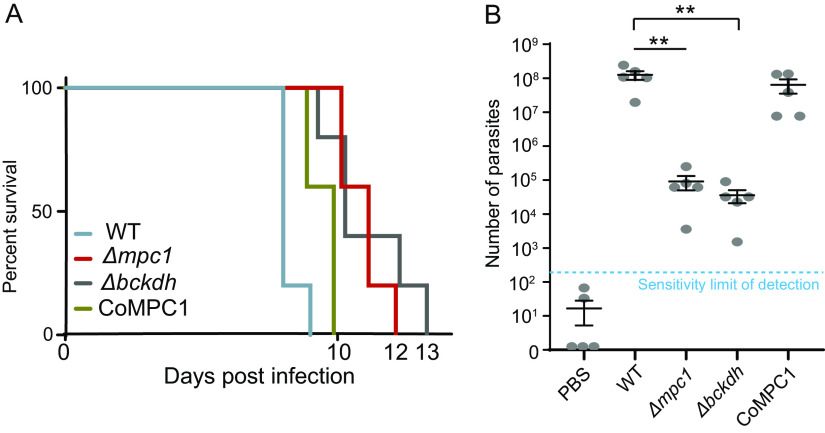
Virulence and proliferation of MPC-deficient mutants *in vivo*. (A) Survival curves of ICR mice infected with 100 tachyzoites of each strain. (B) Parasite burdens in mouse peritoneal fluids 5 days after infection with 10^5^ tachyzoites of indicated strains (or PBS as control). The number of parasites was determined by quantitative PCR amplifying the Toxoplasma *GAPDH1* gene. Each strain was tested with five mice. The mean values ± SEM are shown. Significance was determined using Student’s *t* test. **, *P* ≤ 0.01.

To investigate the *in vivo* importance of pyruvate metabolism, the Δ*mpc1*, Δ*bckdh*, and CoMPC1 strains were used to infect ICR mice by intraperitoneal (i.p.) injection (100 parasites/mouse), and the mice were monitored for survival. All animals infected with the parental strain died within 9 days, whereas those infected by Δ*mpc1* or Δ*bckdh* mutants survived 3 or 4 days longer, suggesting a mild attenuation of virulence. The CoMPC1 strain was nearly as virulent as the parental parasites, as anticipated (Fig. 6A). Our extended work determined the proliferation of these strains in mice infected with tachyzoites (5 days postinfection with 1 × 10^5^ parasites i.p.). The peritoneal fluids were collected, and the parasite burden was scored by quantitative PCR. The animals infected with the parental or CoMPC1 strain harbored about 1 × 10^8^ cells in the peritoneal fluids (Fig. 6B). However, the parasite burdens in mice infected with the Δ*mpc1* or Δ*bckdh* mutant were close to 1 × 10^5^ (0.1% of the burden in controls), suggesting a strong propagation defect in the indicated mutants.

### MPC1 is required for parasite differentiation and chronic infection.

**FIG 7:**
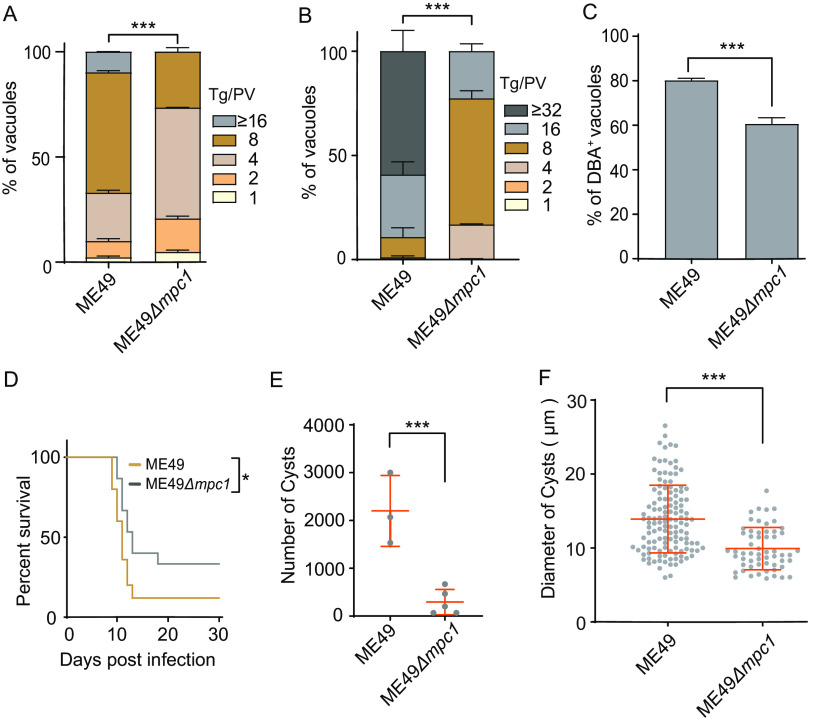
MPC1 inactivation in the type II strain ME49 leads to defects in growth and differentiation. (A and B) Comparison of the replication rates of ME49 and ME49Δ*mpc1* by intracellular replication assays under standard tachyzoite growth conditions (24 h of replication) (A) or alkaline conditions (pH 8.2) (48 h of replication) (B), performed as in the experiment whose results are shown in Fig. 2E. The mean values ± SEM from three independent experiments are shown. Significance was determined using two-way ANOVA. ***, *P* ≤ 0.001. (C) Bradyzoite conversion rates *in vitro* upon growth of parasite in alkaline medium (pH 8.2) for 3 days. Bradyzoites were detected by DBA staining, and total parasites were determined by ALD staining. The mean values ± SEM from three independent assays are shown. Significance was determined using Student’s *t* test. ***, *P* ≤ 0.001. (D) Survival curves of ICR mice infected with the parental strain ME49 or the Δ*mpc1* mutant. Significance was determined using the Gehan-Breslow-Wilcoxon test. *, *P* ≤ 0.05. (E) The numbers of Toxoplasma cysts in the brains of mice infected with ME49 or the Δ*mpc1* mutant. The mean values ± SD from at least 3 surviving mice are shown. Significance was determined using Student’s *t* test. ***, *P* ≤ 0.001. (F) The diameters of cysts from the experiment whose results are shown in panel E. The mean values ± SD from about 50 cysts are shown. Significance was determined using Student’s *t* test. ***, *P* ≤ 0.001.

Finally, we determined the role of MPC in the onset of chronic infection. In this regard, we deleted MPC1 in a type II strain, ME49, that is competent for bradyzoite formation *in vivo* and *in vitro*. Although the ME49Δ*mpc1* mutant was viable and could be maintained in culture, it proliferated much more slowly under normal (tachyzoite) growth and alkaline (bradyzoite induction at pH 8.2 under ambient CO_2_) conditions (Fig. 7A to B). When parasites were cultured in an alkaline medium for 3 days and then stained with Dolichos biflorus agglutinin-fluorescein isothiocyanate (DBA-FITC) to monitor the formation of encysted bradyzoites, the ME49Δ*mpc1* mutant was less efficient in forming DBA-positive (DBA^+^) bradyzoites (Fig. 7C). We also tested the impact of MPC1 deletion on cyst development *in vivo*. The ME49 and ME49Δ*mpc1* strains were used to infect ICR mice (100 tachyzoites/mouse), and the survival of animals was observed for 30 days. The mice infected with the mutant had a mortality rate of 66.7%, compared to 88% with the parental strain, suggesting decreased virulence upon the deletion of MPC1 (Fig. 7D). When the cyst burden in the brains of surviving animals was analyzed on day 30, the Δ*mpc1* mutant was found to have formed nearly 10 times fewer cysts than the ME49 strain (Fig. 7E). Also, the mutant produced much smaller cysts as determined by the diameters of cysts (Fig. 7F). These results demonstrate that MPC1 is required for bradyzoite development both *in vitro* and *in vivo*.

## DISCUSSION

**FIG 8:**
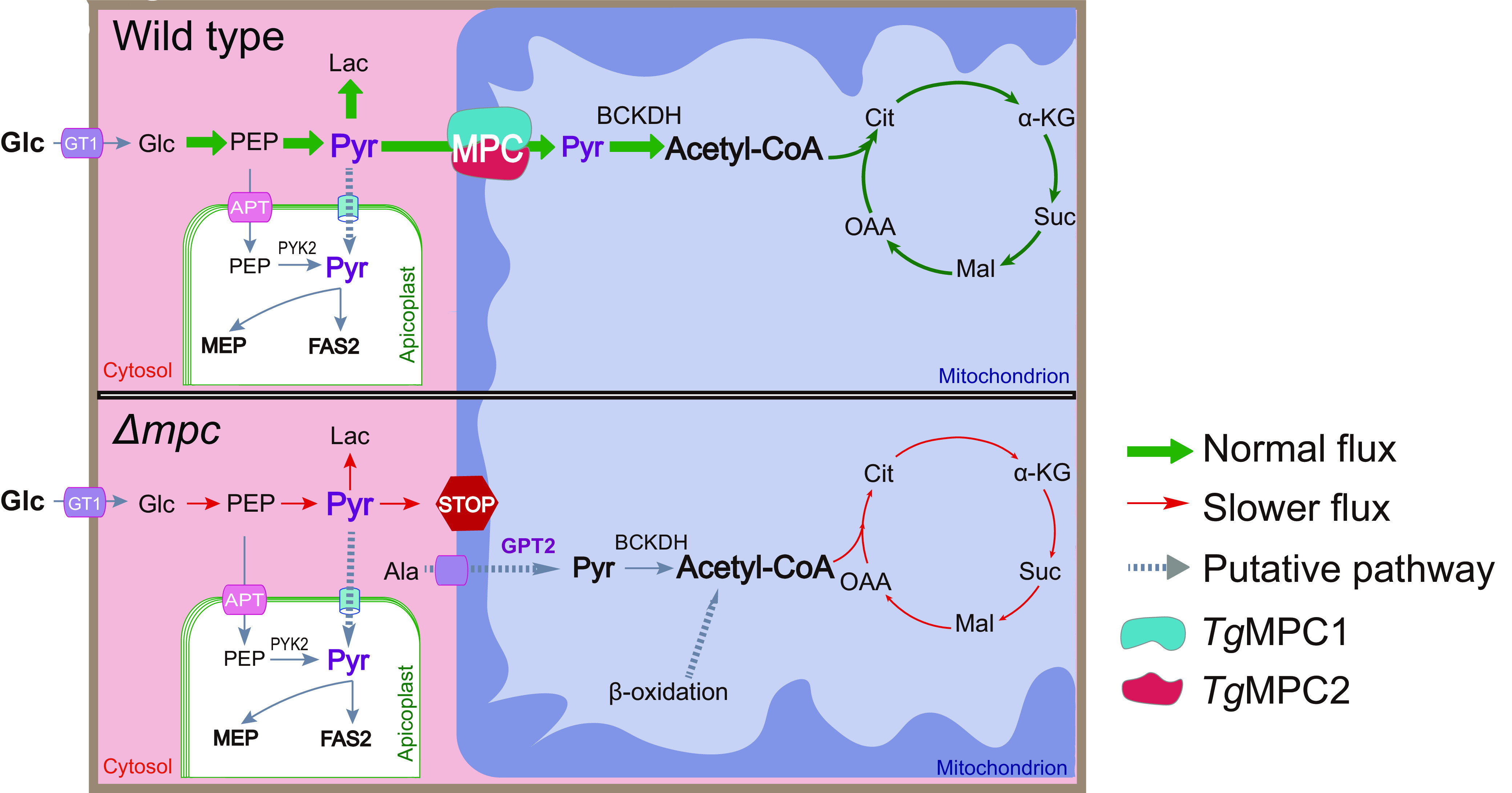
Model for glucose-driven carbon metabolism with or without mitochondrial pyruvate carrier. Glc, glucose; PEP, phosphoenolpyruvic acid; Pyr, pyruvate; Lac, lactate; Ala, alanine; Cit, citrate; α-KG, α-ketoglutaric acid; Suc, succinate; Mal, malate; OAA, oxaloacetic acid. MEP, mevalonate pathway; FAS2, type II fatty acid synthesis pathway; BCKDH, branched-chain ketoacid dehydrogenase; GPT2, glutamic-pyruvic transaminase 2.

Pyruvate is produced in the cytosol and utilized by several metabolic pathways in different organelles of T. gondii. This study aimed to examine the role of pyruvate metabolism in the parasite’s mitochondrion. We identified the mitochondrial pyruvate carrier in T. gondii, comprising MPC1 and MPC2 proteins highly conserved across eukaryotes. Our results from deleting the genes individually or in combination show that both subunits are needed for optimal parasite growth. Mutants lacking functional MPC experienced decreased flux through glycolysis and the TCA cycle, as well as reduced incorporation of glucose-derived carbons into acetyl-CoA (Fig. 8). These metabolic changes were similar to what was observed in the Δ*bckdh* mutant. On the other hand, tachyzoites lacking MPC or BCKDH were still viable and virulent. These observations emphasize the extreme flexibility of tachyzoites to thrive and establish parasitism in various nutritional milieus.

The dispensability of MPC also suggests alternative sources for pyruvate or acetyl-CoA in the mitochondrion of T. gondii (Fig. 8). Unlike yeast and mammalian cells, which can utilize BCAAs and fatty acids to make acetyl-CoA and thereby maintain normal growth in the absence of MPC (26), the tachyzoite mutants of T. gondii are impaired even under nutrient-rich conditions. Toxoplasma does require BCAAs (leucine, isoleucine, and valine) for growth; however, they are probably not a significant source for acetyl-CoA synthesis in the mitochondrion of tachyzoites, because deletion of the *BCAT* gene, which is involved in BCAA degradation, did not noticeably affect tachyzoite growth (27). In addition, the Δ*mpc2-Δbcat* double mutant phenocopies the Δ*mpc2* mutant, suggesting that BCAAs are not used for acetyl-CoA production in the tachyzoite mitochondrion and are probably needed as essential amino acids for protein synthesis. In mammalian cells, alanine can also be transported into mitochondria and then converted to pyruvate by glutamic-pyruvic transaminase 2 (GPT2) in the mitochondrial matrix (28–30). GPT2 homologs can be found in the Toxoplasma genome, but their contribution to acetyl-CoA production and parasite growth deserves further exploration. Likewise, β oxidation of fatty acids as a possible source of mitochondrial acetyl-CoA has not been investigated in tachyzoites. However, the lack of canonical mitochondrial acyl-carnitine/carnitine carriers and low expression of β oxidation proteins in tachyzoites rule out a contribution of fatty acids to acetyl-CoA synthesis (31).

The tolerance of MPC deletion in Toxoplasma brings into question the role of the TCA cycle. Acetyl-CoA not only fuels energy production but is also required for the acetylation of diverse biomolecules, including proteins, DNA, and RNA. It is currently unknown what underlies the poor growth of the Δ*mpc* and Δ*bckdh* mutants. T. gondii harbors additional enzymes in other organelles to produce acetyl-CoA for different reactions (32), which include acetyl-CoA synthetase (ACS) and ATP citrate lyase (ACL) in the cytosol (32) and apicoplast-localized PDH (33). Disruption of these enzymes is not lethal except for the simultaneous deletion of ACS and ACL. Loss of ACS and ACL causes hypoacetylation of nucleocytosolic and secretory proteins, whereas the Δ*bckdh* strain displays hypoacetylation of mitochondrial proteins, suggesting acetylation of organelle proteins by a locally produced pool of acetyl-CoA. Whether the change in mitochondrial protein acetylation is responsible for the growth defect of the Δ*mpc* and Δ*bckdh* mutants merits further investigation.

Another major role of the mitochondrial acetyl-CoA is to drive energy production. T. gondii has a fully functional TCA cycle (12, 13), but its physiological role is not well defined. Conditional depletion of the succinyl-CoA synthetase causes only a 30% reduction in the parasite’s growth (34), and the lack of a more severe growth defect suggests that the TCA cycle is not essential in tachyzoites. Notably, the Δ*mpc* and Δ*bckdh* strains phenocopy the depletion of succinyl-CoA synthetase, endorsing a nonessential role of the TCA cycle. Likewise, six of the eight TCA cycle enzymes in Plasmodium falciparum can be deleted without impairing the erythrocytic development of the malaria parasites (35). MacRae et al. reported that sodium fluoroacetate (NaFAc), a potent inhibitor of aconitase, can abolish the plaque growth of T. gondii tachyzoites, suggesting a critical role of the TCA cycle (13). The authors also described a GABA shunt in T. gondii that could produce succinate from glutamine as a carbon source independently of succinyl-CoA synthetase. It is unclear whether inhibition of aconitase is the only cause of growth arrest by NaFAc or if it exerts a pleiotropic effect by inhibiting other pathways, including mammalian enzymes. The physiological roles of the TCA cycle in T. gondii, therefore, warrant systematic investigation using a chemogenetic approach.

## MATERIALS AND METHODS

### Parasite strains and cell cultures.

The type I (RHΔ*hxgprt* and RH Δ*ku80*), type II (ME49), and subsequent derivative strains of T. gondii were propagated in human foreskin fibroblast (HFF) cells (ATCC, USA) as described elsewhere (36). HFF cells were cultured in Dulbecco’s modified Eagle’s medium (DMEM) supplemented with 10% fetal bovine serum (FBS; Life Technologies, USA), 100 μg/mL streptomycin, and 5 mM l-glutamine. The Δ*mpc1* and Δ*mpc2* mutants of S. cerevisiae and the *pJR3455A* vector for the ectopic expression of MPC proteins (37) were provided by Jared Rutter (University of Utah, USA).

### Construction of plasmids.

The primers used in this study (Table S1) were synthesized by Tsingke Biotechnology (China) and Life Technologies (Germany). Locus-specific CRISPR plasmids were generated by replacing the UPRT-targeting guide RNA (gRNA) in *pSAG1*::*Cas9-U6*::*sgUPRT* with the target-specific gRNAs. The donor plasmids used to delete *MPC1*, *MPC2*, or *BCKDH* genes were generated by cloning the corresponding 5′ and 3′ homology arms and the drug selection marker (DHFR*) into *pUC19*, using the ClonExpress II one-step cloning kit (Vazyme Biotech, China) (20). The *pTub*::*MPC1-HA-CAT* construct for MPC1 complementation (CoMPC1) was made by replacing *Tg*LDH1 in *pCom-LDH1* (38). The yeast expression constructs in the *pJR3455A* vector were generated by restriction cloning of the corresponding open reading frames. The PCR amplicons and vector were digested by XbaI and XhoI and ligated using T4 DNA ligase (TaKaRa, Japan), followed by transformation of E. coli strain XL1b and screening of positive clones. All constructs were verified by sequencing before downstream applications.

### MPC complementation in S. cerevisiae.

The empty vector (*pJR3455A*) and indicated MPC constructs (*Sc*MPC1/2 and *Tg*MPC1/2) were transformed into the *ScΔmpc1* or *ScΔmpc2* mutants following a protocol for yeast culture and transformation described previously (39). Briefly, the *ScΔmpc1* or *ScΔmpc2* mutant was grown in 2% yeast extract, 1% peptone, and 2% glucose. Following transformation with the plasmids described above, all yeast strains were cultured in synthetic dropout (uracil-free) minimal medium (0.67% yeast nitrogen base; Difco) supplemented with appropriate amino acids and 2% glucose. The transfectants were cloned on selective plates and tested for growth complementation in the presence or absence of leucine and valine (30°C for 3 days on plates or up to 32 h in liquid culture).

### Construction of transgenic parasites.

To insert an HA tag at the C terminus of MPC1 and MPC2, the cassette containing the smHA epitope and *DHFR* * selection marker was amplified using the *pSL24m-Linker-smFP-DHFR-LoxP-T7* plasmid (22) and primers with arms (50 bp) homologous to the target genes. The amplicons were cotransfected into purified tachyzoites of the RH Δ*ku80* strain along with the corresponding locus-specific CRISPR plasmid. Transfected parasites were selected with 1 μM pyrimethamine (Sigma-Aldrich, USA), and stable transfectants were examined by immunofluorescence assay. To construct the Δ*mpc1*, Δ*mpc2*, and Δ*bckdh* mutants, corresponding homology templates (MPC1::*DHFR*, MPC2::*DHFR*, or BCKDH::*DHFR*) were transfected into tachyzoites of the RHΔ*hxgprt* strain together with gene-specific gRNA constructs. Transgenic parasites were selected with 1 μM pyrimethamine and cloned by limiting dilution, followed by diagnostic PCRs (PCR1, PCR2, and PCR3, as indicated in the figures). The Δ*mpc1*-Δ*mpc2* double deletion mutant was constructed in two steps. First, the DHFR* selection marker (flanked by *loxP* sites) in the Δ*mpc2* strain was removed by transfecting a plasmid expressing the Cre recombinase (*pmin-Cre-eGFP*) (40). The Δ*mpc2* clones lacking the DHFR* marker were used to further delete the MPC1 gene, as described above. The complementation strain *CoMPC1* was constructed by inserting an MPC1 expression cassette at the *UPRT* locus of the Δ*mpc1* strain. Briefly, the *pTub*::*MPC1-HA-CAT* amplicon was generated from the *pTub*::*MPC1-HA-CAT* plasmid and transfected into tachyzoites along with the *pSAG1*::*Cas9-U6*::*sgUPRT* plasmid. Subsequently, the parasites were selected with 30 μM chloramphenicol (Sigma-Aldrich, USA) (38).

### *In vitro* phenotyping of parasite mutants.

The plaque and replication assays to determine the overall growth and proliferation fitness of tachyzoites were performed following the protocols reported previously (41). To evaluate the bradyzoite differentiation efficiencies of the ME49 and ME49Δ*mpc1* strains, parasites were cultured for 3 days in alkaline RPMI 1640 medium supplemented with 1% FBS and 50 mM HEPES (pH 8.2, ambient CO_2_). Samples were fixed by 4% paraformaldehyde, permeabilized in 0.1% Triton X-100 (Sigma-Aldrich, USA), and then stained with Hoechst 33342 (Beyotime, China), FITC-conjugated Dolichos biflorus agglutinin (DBA-FITC; Vector Laboratories, USA), and rabbit anti-ALD (fructose-bisphosphate aldolase) antibody (provided by David Sibley, Washington University, St. Louis, MO, USA). The formation of bradyzoites was calculated by the ratio of DBA-FITC-positive versus ALD-expressing vacuoles.

### Parasite proliferation and virulence assays in mice.

To assess the parasite virulence, 8-week-old female ICR mice were infected with purified tachyzoites through intraperitoneal (i.p.) injection. Each strain was tested in 5 to 25 mice (depending on the strain tested) with a dose of 100 tachyzoites/animal (38). The mice were monitored daily for their survival over a period of 30 days. To determine the proliferation of parasites, 1 × 10^5^ tachyzoites of indicated strains were injected (i.p.) into mice. Five days postinfection, the peritoneal liquid was examined for the parasite burden by quantitative PCR (qPCR) of the T. gondii glyceraldehyde-3-phosphate dehydrogenase 1 (*Tg*GAPDH1) (primers in Table S1). All animal experiments were approved by the ethical committee of Huazhong Agricultural University (permit number HZAUMO-2019-095).

### Immunofluorescence imaging.

Indirect immunofluorescence assays were performed according to previously described protocols (42). The following primary antibodies were used: mouse anti-HA (Medical & Biological Laboratories Co., Japan), rabbit anti-HSP60, and rabbit anti-*Tg*ALD antibodies. Alexa Fluor 488- or Alexa Fluor 594-conjugated secondary antibodies (Life Technologies, USA), Hoechst 33342 (Beyotime, China), and FITC-conjugated Dolichos biflorus agglutinin (DBA-FITC) (Vector Laboratories, USA) were deployed as indicated to visualize the signals. Parasites were imaged with the FV1000 LSCM confocal laser scanning microscope (Olympus, Japan).

### Metabolic labeling and measurements.

To monitor the carbon flux through glycolysis and the TCA cycle, 3 × 10^7^ tachyzoites were incubated in DMEM containing 8 mM [^13^C_6_]glucose (37°C for 4 h). Subsequently, the parasites were washed once with glucose-free DMEM and twice with phosphate-buffered saline (PBS) and then resuspended in 80% methanol. Samples were lysed by ultrasonication (5 cycles of 1 min each with 1-min intervals on an ice-water interface) in a bath sonicator, incubated for 30 min at −20°C, and centrifuged (15,000 × *g* for 15 min at 4°C). The supernatant of each sample was collected for further analysis to determine the incorporation of ^13^C into glycolytic and TCA cycle intermediates. The supernatant (1 mL) was evaporated to dryness and then reconstituted in 50 μL of 50% aqueous acetonitrile (1:1 [vol/vol]) prior to ultra-high-performance liquid chromatography–high-resolution mass spectrometry (UHPLC-HRMS) analysis.

Chromatographic separation was performed on a ThermoFisher UltiMate 3000 UHPLC system using a Waters BEH amide column (2.1 mm by 100 mm, 1.7 μm). The injection volume was 2 μL, and the flow rate was adjusted to 0.35 mL/min. The mobile phases consisted of water with 15 mM ammonium acetate (pH 8.5, phase A) and acetonitrile in water (90:10 [vol/vol], phase B). A linear-gradient elution with the following program was performed: 0 to 2 min, 90% B; 14 min, 75% B; 15 min, 65% B; 15.2 to 16.9 min, 50% B; 17 to 20 min, 90% B. Eluents were analyzed on a ThermoFisher Q Exactive hybrid quadrupole-Orbitrap mass spectrometer (QE) set in heated electrospray ionization-negative (HESI^−^) mode. The spray voltage was set to 3,500 V, while the capillary and probe heater temperatures were kept at 250°C and 300°C. The sheath and auxiliary gas flow rates and the S-lens radiofrequency (RF) level were adjusted to 35 arbitrary units (AU), 10 AU, and 50 AU, respectively. The instrument was operated in a full-scan high-resolution mode (70,000 full width half maximum [FWHM]; *m/z*, 200) in a range of 70 to 1,050 *m/z* with the automatic gain control (AGC) target setting at 1 × 10^6^.

Acetyl-CoA was analyzed by LC-MS, performed using the 6500 plus Qtrap mass spectrometer (AB Sciex, USA) coupled to the Acquity UPLC H class system (Waters, USA). An Acquity UPLC HSS T3 column (2.1 by 100 mm, 1.8-μm particle size; Waters) was employed, using water with 5 mM ammonium acetate as mobile phase A and methanol as mobile phase B. The linear gradient was set as follows: 0 min, 0% B; 1.5 min, 0% B; 6 min, 95% B; 7.4 min, 95% B; 7.5 min, 0% B; and 10 min, 0% B (flow rate, 0.3 mL/min). The column chamber and sample tray were kept at 35°C and 10°C, respectively. The mass data were acquired in multiple reaction monitor (MRM) mode for acetyl-CoA, acetyl-CoA (*M2) and acetyl-CoA (*M3) with transitions of 810/303, 812/305 and 813/306 in positive mode. The ion transitions were optimized using the chemical standards. The nebulizer gas (Gas1), heater gas (Gas2), and curtain gas were set at 55, 55, and 30 lb/in^2^, respectively. The ion spray voltage was 5,500 V, and the optimal probe temperature was set at 550°C. The Sciex OS1.6 software was applied for metabolite identification and peak integration.

### Protein sequence analysis.

Protein sequences were retrieved from NCBI (https://www.ncbi.nlm.nih.gov). The multiple-sequence alignment was performed using ClustalX2 and the following sequences: T. gondii MPC1, accession number EPR60295.1; S. cerevisiae MPC1, NP_011435.1; P. falciparum MPC1, XP_001350132.1; Mus musculus MPC1, NP_001351848.1; Homo sapiens MPC1, NP_057182.1; Arabidopsis thaliana, NP_001078606.1; Neospora caninum MPC1, XP_003884618.1; Drosophila takahashii MPC1, XP_044250136.1; T. gondii MPC2, XP_002367709.1; Arabidopsis thaliana MPC2, KAG7615987.1; S. cerevisiae MPC2, NP_012032.1; Homo sapiens MPC2, NP_001137146.1; Neospora caninum MPC2, XP_003882333.1; P. falciparum MPC2, XP_001348845.2; Mus musculus MPC2, NP_081706.1; Drosophila takahashii MPC2, XP_016996855.2.

### Data presentation and statistics.

All assays were performed at least three times independently, unless stated otherwise. Statistical analyses were executed in GraphPad Prism 8.0.2 (GraphPad Software, Inc., USA) using two-way analysis of variance (ANOVA) with the Bonferroni posttest or Student’s *t* test. The data in graphs depict the mean values ± standard errors of the means (SEM) or standard deviations (SD), as indicated in figure legends (*, *P* ≤ 0.05; **, *P* ≤ 0.01; ***, *P* ≤ 0.001). The plaque and immunofluorescence images shown are representative of multiple experiments.

### Data availability.

All data generated or used in this study are presented in the paper and the supplemental material.
