# Supplementary material for: The Mitochondrial Pyruvate Carrier Coupling Glycolysis and the Tricarboxylic Acid Cycle Is Required for the Asexual Reproduction of Toxoplasma gondii
Source: Microbiol Spectr. 2023 Mar 15;11(2):e05043-22. doi: 10.1128/spectrum.05043-22 (PMC10100952; doi:10.1128/spectrum.05043-22)
Supplement: Supplemental file 1 — Supplemental material. Download spectrum.05043-22-s0001.pdf, PDF file, 1.9 MB [file spectrum.05043-22-s0001.pdf]

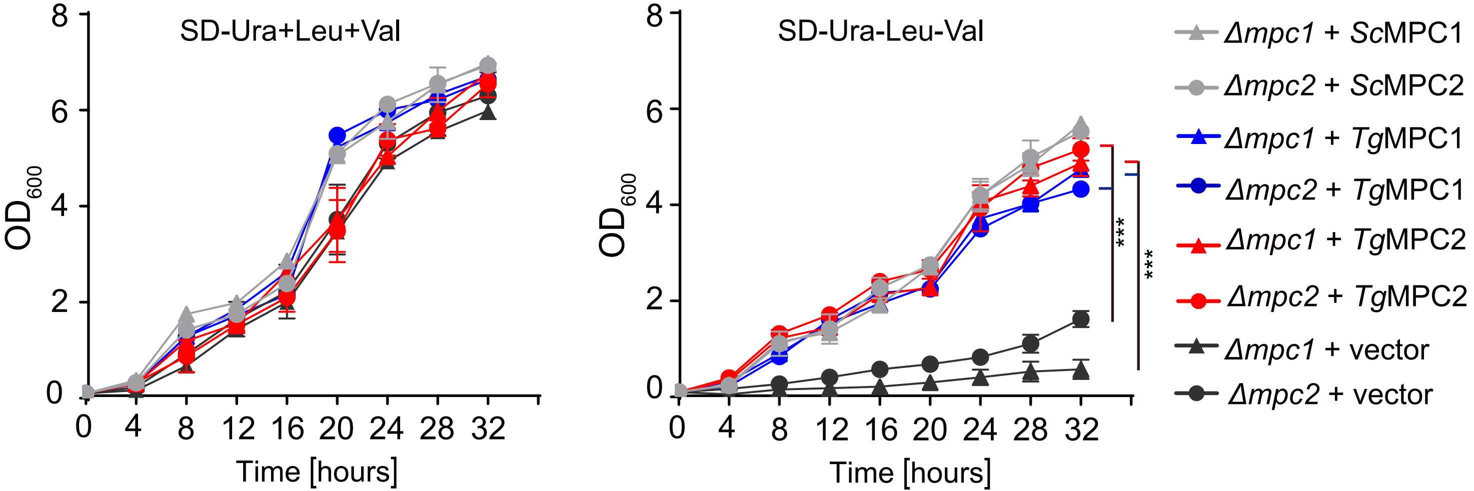

Figure S1. Liquid assay testing the growth of yeast *Δmpc1* or *Δmpc2* mutants expressing parasite MPC1 or MPC2 in medium with (+) or without (-) valine and leucine. Indicated strains were cultured in 10 ml medium and 1 ml culture from each sample was taken out every 4 hours for OD_{600} measurement. Means ± SEM of three independent experiments. ****p* ≤ 0.001, two-way ANOVA.

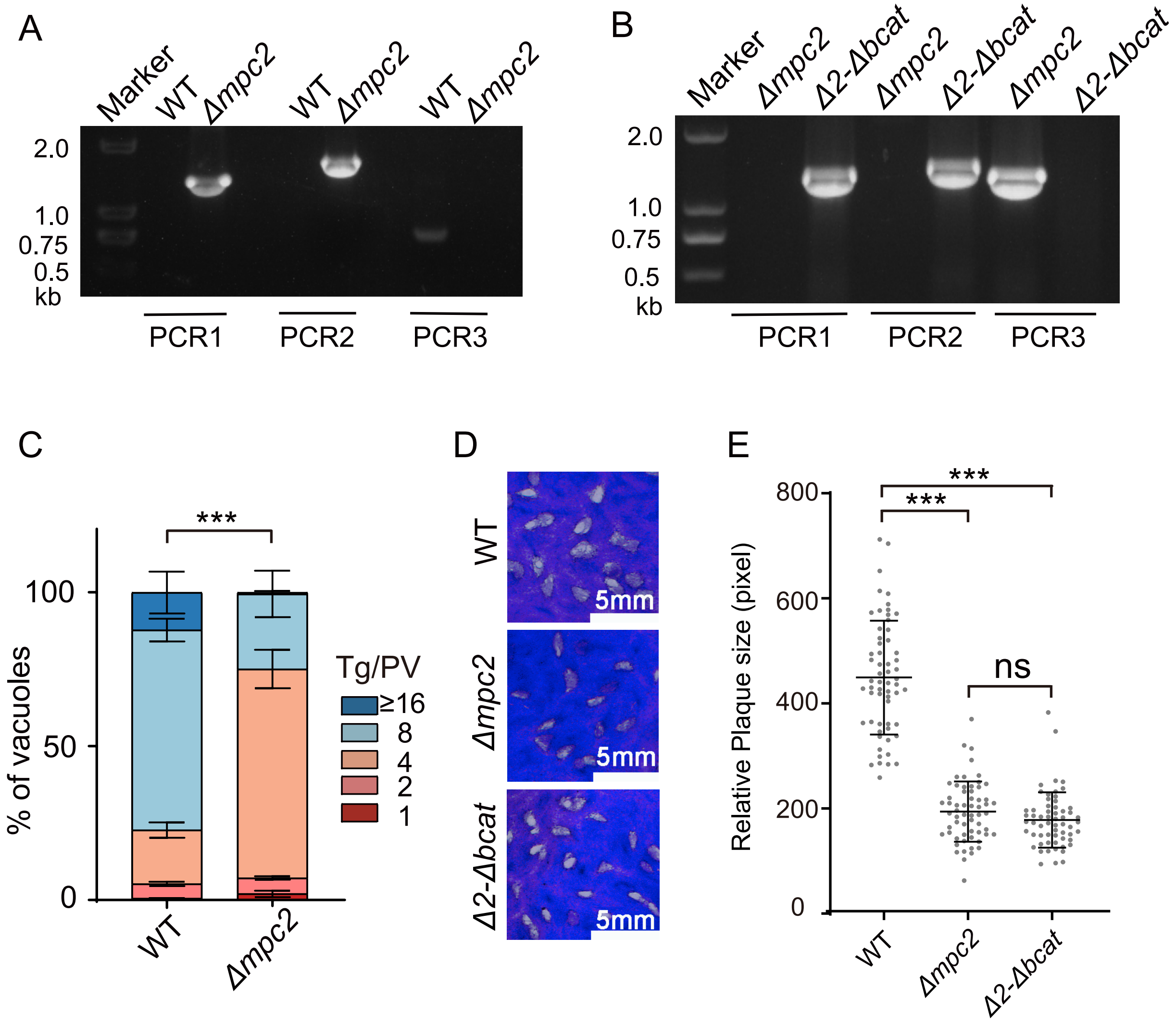

Figure S2. Mutant lacking both MPC2 and BCAT displayed similar growth defects as the *Δmpc1* mutant. A, Diagnostic PCRs on a MPC2 deletion mutant, which was generated by replacing *MPC2* in RH strain with *DHFR**, using the same strategy described in figure 2a. B, Diagnostic PCRs on a *Δmpc2-Δbcat* mutant, which was generated by replacing BCAT in the *Δmpc2* (the one with *DHFR** selection marker deleted) mutant with *DHFR**, using the same strategy described in figure 2A. C, a 24-hour intracellular replication assay comparing the proliferation rates of indicated strains, as done in figure 2E. ****p* ≤ 0.001, two-way ANOVA. D&E, Plaque assays comparing the growth of the *Δmpc2* and *Δmpc2-Δbcat* mutants to that of RH, as performed in figure 2 F-H. ****p* < 0.001, Student's t-tests.

Table S1. Primers used in this study

Primers	Sequences	Used for
Konckout-MPC1-gRNA-Fw	GGCAGCGGCGAAAGGTGCCGGTTTTAGAGCTAGAAATAGC	Constructing pSAG1-CAS9-sgMPC1 to delete MPC1
Konckout-MPC2-gRNA-Fw	GGGCTCCTGCGCTCAAGTGGGTTTTAGAGCTAGAAATAGC	Constructing pSAG1-CAS9-sgMPC2 to delete MPC2
Konckout-BCKDH-gRNA1-Fw	CACGCTGTTCCGAAGCACTCGTTTTAGAGCTAGAAATAGC	Constructing pSAG1-CAS9-sgBCKDH1
Konckout-BCKDH-gRNA2-Fw	GCGACAGTTACCAAATGGCGGTTTTAGAGCTAGAAATAGC	Constructing pSAG1-CAS9-sgBCKDH2
BCKDH-gRNA2-KpnI-Fw	CGAATTGGGTACCCAAGTAAGCAGAAGCACGCTG	Constructing pSAG1-CAS9-dual-gBCKDH to delete BCKDH
BCKDH-gRNA2-XhoI-Rv	TACCGTCGACCTCGAGGAATTAACCCTCACTAAAGG	Constructing pSAG1-CAS9-dual-gBCKDH to delete BCKDH
Local-MPC1-gRNA-Fw	GCTTCGTCATGGATCTGGTGGTTTTAGAGCTAGAAATAGC	Constructing pSAG1-CAS9-sgMPC1-loc for localization
Local-MPC2-gRNA-Fw	GTGTCACCAGCATCAAGCGAGTTTTAGAGCTAGAAATAGC	Constructing pSAG1-CAS9-sgMPC2-loc for localization
MPC1-HA-loc-Fw	GGAAGCTCAGGTACAACTCGGAGCAACGGACCAAAACCGCGAA TGCTACAGGCTCGACgAGGATGTACCC	Amplifying donor fragment for MPC1 localization
MPC1-HA-loc-Rv	GTAAACCCAGGCCGGTCGCTGCCGTTCTGGGTGTCGCTGCATCG CGTCAGCACCGCTTTCTCAACAGGAA	
MPC2-HA-loc-Fw	TGCACCACCTTGCGGAGAAGGAGAAAGCTTCGTCGGCAGCTCC GTCGTCGGGCTCGACgAGGATGTACCC	Amplifying donor fragment for MPC2 localization
MPC2-HA-loc-Rv	ACTAACACAACTTCTAACGGAAAACTGCTCCAGCAACTTCAAGA	

	CCACAGCACCGCTTTCTCAACAGGAA	
gRNA-Rv	AACTTGACATCCCCATTTAC	Constructing locus specific CRISPR plasmids
U5-MPC1-Fw	CGACTCACTATAGGGCGAAT TGCGGTATGTCATCAACCAG	Amplifying the 5' homologous arm of *MPC1* to construct pMPC1::DHFR
U5-MPC1-Rv	GATGTCTTCTGCGCGGGTTG CACAGTCGGAAAGACACTCAC	
U5-MPC2-Fw	CGACTCACTATAGGGCGAAT CATGTTCCCTGTTTCCTTTCGAG	Amplifying the 5' homologous arm of *MPC2* to construct pMPC2::DHFR
U5-MPC2-Rv	GATGTCTTCTGCGCGGGTTG TCTCCTTCGCTCTCGCCTC	
U5-BCKDH-Fw	CGACTCACTATAGGGCGAATTCTCCGGTTCCCTGACTCTC	Amplifying the 5' homologous arm of *BCKDH* to construct pBCKDH::DHFR
U5-BCKDH-Rv	GATGTCTTCTGCGCGGGTTGAGTCGATGACAAGCACGAAAC	
U3-MPC1-Fw	GCCACAAGTTCAGCGTGTCC TTCTGGCTGTGACTGGTGTG	Amplifying the 3' homologous arm of *MPC1* to construct pMPC1::DHFR
U3-MPC1-Rv	GCTATGACCATGATTACGCC ATGGCAACAGCACTCAAACC	
U3-MPC2-Fw	GCCACAAGTTCAGCGTGTCCAGCATCAAGCGACGGATTTC	Amplifying the 3' homologous arm of *MPC2* to construct pMPC2::DHFR
U3-MPC2-Rv	GCTATGACCATGATTACGCCTCTCGGGAACAGCGGTCATC	
U3-BCKDH-Fw	GCCACAAGTTCAGCGTGTCCTCGCTGCTTTCGCGAGTTTG	Amplifying the 3' homologous arm of *BCKDH* to construct pBCKDH::DHFR
U3-BCKDH-Rv	GCTATGACCATGATTACGCCGTTAACGACCCTGCGGCACTC	
DHFR-Fw	CAGGCTGTAAATCCCGTGAG	Amplifying DHFR to construct pMPC1/MPC2/BCKDH::DHFR
DHFR-Rv	GATTCCGTCAGCGGTCTGTC	
pUC19-vector-Fw	GGCGTAATCATGGTCATAGC	Amplifying the pUC19 vector fragment to construct pMPC1/MPC2/BCKDH::DHFR
pUC19-vector-Rv	CTCGAATTCACTGGCCGTCG	
CAT-Fw	GAGGTCGACGGTATCGATAA	Amplifying CAT to construct pMPC2::CAT
CAT-Rv	CGCTCTAGAACTAGTGGATC	
MPC1-PCR1-Fw	TCTAGGATGACGGACTACACC	PCR1 of *Δmpc1::DHFR*
MPC1-PCR1-Rv	CCTAGGGTCAAGTGGATCTTGG	
MPC1-PCR2-Fw	TCGGTAGCGACGAGGATATG	PCR2 of *Δmpc1::DHFR*
MPC1-PCR2-Rv	AGAAGTGCCCGAGATTCTG	
MPC1-PCR3-Fw	TGTCGGCCTGTTTGTTGC	PCR3 of *Δmpc1::DHFR*

MPC1-PCR3-Rv	TCGCGTTACCGTTATGTAGC	
MPC2-PCR1-Fw	ACGCATGATAAGACCTCTCC	PCR1 of *Δmpc2::DHFR*
MPC2-PCR1-Rv	CCTAGGGTCAAGTGGATCTTGG	
MPC2-PCR2-Fw	TCGGTAGCGACGAGGATATG	PCR2 of *Δmpc2::DHFR*
MPC2-PCR2-Rv	GCTAGTGTATGGTACCGAGT	
MPC2-PCR3-Fw	TTCGCTCCCTACCTCCAGGC	PCR3 of *Δmpc2::DHFR*
MPC2-PCR3-Rv	TGACTGCTGAGCAACGGAAG	
BCKDH-PCR1-Fw	CTCATGGTGAATGCGTTCTC	PCR1 of *Δbckdh::DHFR*
BCKDH-PCR1-Rv	TTTGTCGGAATTCTATAACTTCG	
BCKDH-PCR2-Fw	CACGGACGAATCCAGATGGAG	PCR2 of *Δbckdh::DHFR*
BCKDH-PCR2-Rv	AGTGCTGGTTGTTCGCGTG	
BCKDH-PCR3-Fw	ACCGACATGAACATCAGCAAC	PCR3 of *Δbckdh::DHFR*
BCKDH-PCR3-Rv	CGGTACTGGCAGAAGAGATGATC	
MPC1-CDS-Fw	GATCTAAAATGGTGAGCAAG ATGTGGTTGACTCTTGGTCG	Amplifying MPC1 from cDNA of RH strain
MPC1-CDS-Rv	TCTGGAACATCGTAAGGATATGTAGCATTCGCGGTTTTG	
Comp-vector-Fw	TATCCTTACGATGTTCCAGATT	Amplifying the tubulin promoter, CAT cassette and smHA from pCom-LDH1
Comp-vector-Rv	CTTGCTCACCATTTTAGATC	
TgMPC1-XbaI-F	CTCATCTCTAGAATGTGGTTGACTCTTGGTCG	Constructing pJR3455A-MPC1
TgMPC1-3xFLAG-XhoI-R	TCATCCTCGAGTTACTTGTCATCGTCATCCTTGTAATCGATATCATGATCTTTATAATCACCGTCATGGTCTTTGTAGTCTGTAGCATTCGCGGTTTTGG	
TgMPC2-XbaI-F	CTCATCTCTAGAATGGCATCTGTGGTCCACAAAATCTTCTTCC	Constructing pJR3455A-MPC2
TgMPC2-3xFLAG-XhoI-R	CTCATCCTCGAGTCACTTGTCATCGTCATCCTTGTAATCGATATCATGATCTTTATAATCACCGTCATGGTCTTTGTAGTCCGACGACGGAGCTGCCGA	

GAPDH1-qPCR-F	TGAAGGGAATCATCAGCTACAC	qPCR for parasite load determination
GAPDH1-qPCR-R	TGTCAAACACGGAGGAGAAC	
